# A phase II study of concurrent chemoradiotherapy and erlotinib for inoperable esophageal squamous cell carcinoma

**DOI:** 10.18632/oncotarget.9809

**Published:** 2016-06-03

**Authors:** Chuanhua Zhao, Li Lin, Jianzhi Liu, Rongrui Liu, Yuling Chen, Feijiao Ge, Ru Jia, Yang Jin, Yan Wang, Jianming Xu

**Affiliations:** ^1^ Department of GI Oncology, 307 Hospital of PLA, Academy of Military Medical Sciences, Beijing, China

**Keywords:** esophageal cancer, epidermal growth factor receptor, chemoradiotherapy, erlotinib, paclitaxel

## Abstract

Cisplatin-based concurrent chemoradiotherapy for patients with unresectable, locally advanced esophageal squamous cell carcinoma (ESCC) is associated with significant toxicities that are often intolerable. Prognosis for this subgroup of patients remains poor, and new therapeutic approaches are urgently needed. We investigated the efficacy and safety of paclitaxel combined with erlotinib and concurrent radiotherapy in patients with inoperable ESCC. Erlotinib (150 mg) was administered daily for 60 days beginning at the start of radiotherapy, and paclitaxel (45 mg/m^²^) was administered weekly along with intensity modulated conformal radiotherapy (60 Gy in 30 fractions). The median follow-up time was 21 months. The associations between EGFR and VEGF expression and treatment outcome were evaluated. Among the 21 patients treated, the overall response rate (CR + PR) was 85.6%. The median LPFS, PFS and OS were: 17.5, 14.3, and 22.9 months, respectively. Treatment-related grade 3 toxicities included esophagitis (two patients) and hypoleukemia (one patient). Grade 4 pulmonary toxicity was observed in one patient. Patients expressing EGFR had longer PFS, while those expressing VEGF or with a history of smoking had worse outcomes. Weekly paclitaxel combined with erlotinib and concurrent radiotherapy shows promise as an effective, tolerated regimen for patients with inoperable ESCC.

## INTRODUCTION

Esophageal cancer is the eighth most common cancer worldwide and the fourth most common in China [1]. At the time of diagnosis, two-thirds of patients will have tumors that are considered inoperable due to comorbidities or tumor extension [2]. Concurrent chemoradiotherapy (CRT) based on cisplatin and fluorouracil (PF) is the standard treatment for patients with inoperable, locally advanced esophageal squamous cell carcinoma (ESCC) [3]. This therapeutic approach reduces mortality somewhat but at a cost of increased toxicity, and the 5-year overall survival (OS) rate remains less than 20% [4]. Clearly, new therapeutic agents and treatment strategies are urgently needed for this subgroup of patients.

Paclitaxel is a widely used chemotherapeutic agent that has demonstrated promising antitumor activity in patients with esophageal cancer [5]. A study comparing weekly paclitaxel with a PF regimen for ESCC patients undergoing synchronous combined chemotherapy and radiotherapy (RT) reported similar immediate curative effects and 1, 3, and 5-year survival rates in the two groups, though greater digestive toxicity was observed in the PF group [6]. In China, weekly paclitaxel combined with RT is considered a feasible regimen for ESCC patients.

Epidermal growth factor receptor (EGFR) is overexpressed in 30–70% of ESCC cases and is associated with a poor prognosis and inferior response to conventional treatment [7]. Erlotinib is a small molecule inhibitor that reversibly targets EGFR [8]. Several phase I/II trials have evaluated the feasibility of using erlotinib in combination with CRT based on cisplatin [9]. In the present study, we tested whether using erlotinib and paclitaxel instead of more toxic chemotherapeutic agents in combination with concurrent radiotherapy would improve treatment outcomes. This phase II study was designed to evaluate the safety and efficacy of paclitaxel combined with erlotinib and concurrent radiotherapy for the treatment of inoperable ESCC.

## RESULTS

### Patient characteristics

Twenty-one patients were enrolled in the trial between October 2011 and October 2013. The median length of follow-up for patients who survived to the time of analysis was 21 months. The majority of the patients were male (17/21). The median age was 61 years (range, 40–77 years). The most common site of the primary tumor was the upper thoracic esophagus (*n* = 10), followed by the middle thoracic esophagus (*n* = 5), cervical esophagus (*n* = 4), and lower thoracic esophagus (*n* = 2). The majority of patients had stage III disease (20/21). Stage II, IIIA and IIIC primary disease was observed in 1, 13 and 7 patients, respectively. Preexisting cardiovascular disease, cerebrovascular disease, and diabetes were recorded in 3, 2 and 1 patients, respectively. Baseline patient and tumor characteristics are summarized in Table 1.

**Table 1:** Baseline patient characteristics

Characteristic	No. of patients	%
All Patients	21	100
Gender		
Male	17	81
Female	4	19
Age (years)		
≤ 60 years	10	47
60–70 years	7	33
> 70 years	4	20
ECOG PS		
0 and 1	20	95
2	1	5
TNM stage (UICC 2002)		
II	1	5
IIIA	13	62
IIIB	0	0
IIIC	7	33
Location		
Cervical	4	
Upper	10	
Middle	5	
Lower	2	
EGFR mutation (*n* = 10)		
Positive	0	0
Negative	10	100
EGFR expression		
Positive	16	76
Negative	5	24
VEGF expression		
Positive	7	14
Negative	34	66
Smoking status		
Smokers	7	33
Non-smokers	14	67

ECOG PS, Eastern Cooperative Oncology Group performance status; TNM, tumor-node-metastasis; EGFR, epidermal growth factor receptor; VEGF, vascular endothelial growth factor.

### Treatment toxicities

The main treatment toxicities observed in our study are listed in Table 2. The most common side effects were esophagitis in 18 patients (85%), followed by hypoleukemia in 5 patients (23.8%), fatigue in 4 patients (19.0%), pulmonary toxicity in 3 patients (14.2%), and skin rash in 2 patients (9.5%). No patients developed diarrhea or nausea. Radiation pneumonitis was observed in 2 patients (9.5%): one patient developed grade 1 radiation pneumonitis, while the second developed grade 2. The erlotinib dose had to be reduced in one patient. For the same patient, the radiation dose had to be decreased to 40 Gy due to grade 4 pulmonary toxicity (diffuse alveolar hemorrhage), though the patient recovered after treatment [10]. Grade 3 hypoleukemia was observed in one patient (4.7%). No additional toxicities greater than grade 3 and no treatment interruptions were recorded.

**Table 2:** Treatment-related toxicities

	Grade 1 *n* %		Grade 2 *n* %		Grade 3 *n* %		Grade 4 n %	
Esophagitis	7	34	9	52	2	9	0	0
Hypoleukemia	2	10	3	14	1	5	0	0
Fatigue	2	10	2	10	0	0	0	0
Pulmonary toxicities	1	5	1	5	0	0	1	5
Skin rash	1	5	1	5	0	0	0	0
Nausea	0	0	0	0	0	0	0	0
Diarrhea	0	0	0	0	0	0	0	0
Thrombocytopenia	2	10	1	5	0	0	0	0

### Treatment outcomes

Among the 21 patients treated, 8 (38.0%) achieved a complete response (CR), 10 (47.6%) a partial response (PR), and 3 (14.4%) stable disease (SD). No patient showed progressive disease (PD). The median local progression-free survival (LPFS), progression-free survival (PFS), and overall survival (OS) were 17.5 months (95% confidence interval [CI]: 13.9–24.0 months), 14.3 months (95% CI: 12.4–21.9 months), and 22.9 month (95% CI: 16.5–27.2 months), respectively. Two-year LPFS, PFS, and OS were 52.4%, 42.8%, and 67.0%, respectively.

### EGFR mutations and treatment outcomes

Ten patient samples were evaluated for EGFR mutations; however, direct sequencing analysis revealed no mutations in any of the tumor samples, likely due to the low mutation rate reported in ESCC [11]. EGFR mutation analysis was therefore discontinued.

### EGFR/vascular endothelial growth factor (VEGF) expression patterns and treatment outcomes

**Figure 1:**
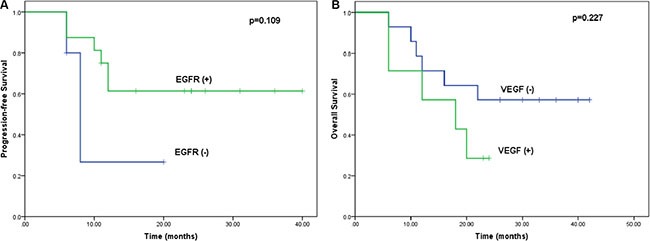
Kaplan-Meier curves comparing OS and PFS among patients with and without EGFR or VEGF expression (**A**) Kaplan-Meier curves for PFS stratified based on EGFR expression (log-rank test: *p* = 0.109). (**B**) Kaplan-Meier curves for OS stratified based on VEGF expression (log-rank test: *p* = 0.227).

EGFR/VEGF expression was evaluated in patients from whom there were sufficient biopsy specimens for immunohistochemical examination. Twenty-one biopsy specimens were evaluated. Tumors from 5 patients exhibited no detectable EGFR expression, whereas those from the remaining 16 patients showed EGFR expression. Tumors from 7 patients showed VEGF expression, while those from the remaining 14 patients had no detectable VEGF expression. Patients with tumors in which EGFR expression was detected tended to have longer PFS, while those with tumors expressing VEGF tended to have worse outcomes, but these differences were not statistically significant (*p* > 0.05; Figure 1A, 1B).

### Smoking status and treatment outcomes

**Figure 2:**
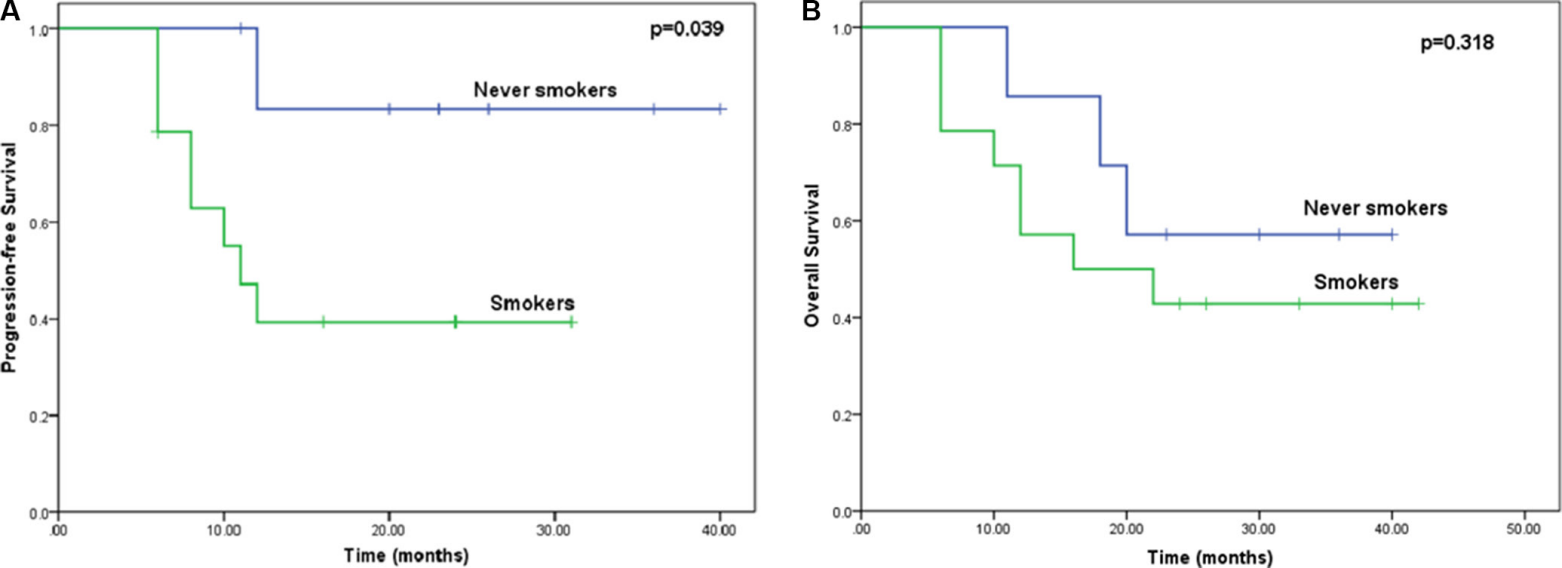
Kaplan-Meier curves comparing OS and PFS among patients with and without a history of smoking (**A**) Kaplan-Meier curves for PFS stratified based on tobacco smoking status (log-rank test: *p* = 0.039). (**B**) Kaplan-Meier curves for OS stratified based on tobacco smoking status (log-rank test: *p* = 0.318).

Of the 21 patients treated, 7 had never smoked. The PFS was significantly better among these patients than among those who smoked (18.1 vs. 11.5 months, *p* < 0.05; Figure 2A), as was OS (24.3 vs. 13.7 months, *p* > 0.05; Figure 2B).

## DISCUSSION

For patients with unresectable, locally advanced ESCC, the prognosis is poor, despite the standard care of concurrent chemoradiotherapy [12]. In an earlier study (RTOG-8501), the 2-year survival rate among ESCC patients was only 36% [13]. In this palliative setting, the efficacy of aggressive combination chemotherapy has been questioned. First, the combination chemotherapy regimen provided effective palliation but resulted in substantial toxicity, with only 59% of patients completing the planned chemoradiation. Second, perhaps due to the high level of toxicity associated with the chemotherapy regimen, the total radiation dose was only 50 Gy in this trial, which is widely considered insufficient for radical treatment of ESCC patients. Similar results were obtained in several other small-sample studies: the response rate, including CR and PR, was approximately 60–70%, while the median survival time was 10–12 months; 2-year local failure and OS were 25–60% and 20–40%, respectively [14]. These results highlight the need to explore new less toxic agents that could potentially replace combined chemotherapy to improve outcomes.

EGFR overexpression correlates with poor prognosis and an inferior response to therapy [18]. This affords a potential opportunity for anti-EGFR agents to improve treatment outcomes. Moreover, a strong rationale may exist for combining erlotinib, an EGFR inhibitor, with RT. First, erlotinib disrupts cell growth pathways and enhances the sensitivity of cells to RT [19]. Second, by exerting a cytoreductive effect and creating a hypoxic environment, RT may enhance the effectiveness of erlotinib [20]. Finally, the toxicities associated with erlotinib, RT and paclitaxel do not overlap, which enables their concomitant use. Hence, it is reasonable to use erlotinib concurrently with chemoradiation in ESCC patients.

In a phase I study to evaluate the combination of erlotinib with cisplatin-based CRT, ESCC patients were assigned to receive a dose of 50, 100, or 150 mg/day erlotinib concurrently with RT and cisplatin or PF [21]. Erlotinib was well tolerated at 150 mg/day. A phase II study found that RT with concurrent PF and erlotinib for patients with locally advanced ESCC had antitumor activity, but was accompanied by significant toxicities. Acute grade 3 toxicities included leukopenia, esophagitis, and skin rash; 4 of 22 patients were unable to complete both cycles of chemotherapy [22]. These initial results suggest that erlotinib has the potential to improve the outcomes of ESCC patients when combined with a more reasonable chemotherapy scheme.

In China, paclitaxel is widely used instead of PF in CRT for esophageal cancer. This regimen reduces toxicities and enables the RT dose to be increased [15]. Traditionally, paclitaxel is administered at 175– 225 mg/m ^2^ over 3 h every 3 weeks. A Cochrane review examining various schedules of paclitaxel infusion in patients with advanced adenocarcinoma concluded that lower doses at more frequent intervals were more effective [16]. Studies comparing metronomic weekly paclitaxel to multidrug combination chemotherapy found that the metronomic scheduling achieved similar clinical benefits with less toxicity [17].

To the best of our knowledge, this is the first study to evaluate the safety and efficacy of erlotinib and paclitaxel combined with RT for ESCC. Concomitant treatment with erlotinib, paclitaxel and RT in locally advanced ESCC patients ineligible for surgery had significant clinical value, and the toxicities were tolerable. The overall response rate (CR + PR) was 85.6%, and the treatment yielded satisfactory 2-year OS and local-regional control. Furthermore, the present study is the first to correlate EGFR and VEGF expression with treatment outcomes. Immunohistochemical examination confirmed that tumors from 16 patients (76%) expressed EGFR. This is consistent with the earlier observation that EGFR is overexpressed in 30–90% of ESCC cases [23]. Our results also indicate that patients with ESCC expressing EGFR had a better PFS than patients with no EGFR expression. By contrast, patients expressing VEGF had a worse OS, though the difference was not significant, perhaps due to the small sample size.

Studies of non-small cell lung cancer (NSCLC) showed that patients with tumors expressing EGFR carrying a mutation were more likely to respond to erlotinib [24]. However, the mechanism of the combined erlotinib and RT differs from erlotinib alone in NSCLC. *In vitro* studies indicate that erlotinib enhances the radiation response at several levels, including cell cycle arrest, apoptosis induction, accelerated cellular repopulation, and DNA damage repair [25]. Erlotinib modulates the radiation response by influencing cell cycle kinetics and apoptosis. Erlotinib in combination with RT reduces the number of cells in S phase while increasing the level of apoptosis and promoting an increase in sensitivity to RT [19]. *In vivo*, erlotinib influences the expression of radiation response genes from several functional classes, including those involved in cell cycle arrest and DNA damage repair [26]. In a preclinical study involving three human cancer cell lines with low, moderate, or very high EGFR expression, the extent of erlotinib-induced radiosensitization was proportional to the EGFR expression level [27]. The cell line expressing very high levels of EGFR exhibited the strongest radioresistance, and treatment with erlotinib increased the extent of G1 arrest and augmented apoptosis in those cells. In addition, the anti-angiogenic agent bevacizumab in combination with erlotinib and RT significantly decreased growth of human VEGF-secreting cell xenografts [26]. These results from preclinical studies highlight the potential benefit of both EGFR and VEGF inhibition.

We found that non-smokers had a better treatment response to erlotinib combined with CRT than smokers, which was also seen in NSCLC patients [28]. Other studies have shown that tobacco smoking can lower the response to erlotinib by reducing its bioavailability by 25% and increasing its clearance rate [29].

Tissues such as skin and gut express EGFR constitutively, which may explain the toxicity associated with erlotinib. In NSCLC studies, skin rash and diarrhea, the major side effects reported, occurred in 47–86% and 30–70% of patients, respectively; however, they were mostly grade 1/2 [30]. In our study, only two patients had grade I skin rashes, and no patients developed diarrhea. Despite the small sample size limiting our ability to draw a definite conclusion, it is noteworthy that erlotinib may have different toxicities in different tumor types, which is consistent with other studies [23].

The present study has several limitations. First, although the results are exciting, this is a single arm, small sample, phase II study. Large, randomized trials will be needed to confirm the benefits of erlotinib administered concurrently with CRT. Second, other molecular markers in addition to EGFR and VEGF should also be identified to predict patient populations that will benefit from erlotinib combined with CRT in ESCC. Third, erlotinib was administered concurrently with radiation; whether other dosing schedules could improve outcomes needs further study.

In conclusion, our data suggest that weekly paclitaxel combined with erlotinib and concurrent radiotherapy shows promise as an effective, tolerated regimen for patients with inoperable ESCC. Our study revealed better outcomes in patients with EGFR expression, no VEGF expression, and no history of smoking. A future randomized study is needed to confirm the benefits of concomitant treatment with erlotinib and chemoradiotherapy in ESCC patients. To predict the TKI response, valuable molecular biomarkers or clinical factors should be exploited in the future.

## MATERIALS AND METHODS

### Study design

We performed an open-label, single arm, phase II trial with patients recruited from our Department of GI Oncology. Patients received CRT with erlotinib (150 mg/day). CRT consisted of paclitaxel 45 mg/m² weekly concurrently with 60 Gy of radiotherapy given in 30 fractions. The primary endpoint was 2-year OS and PFS. LPFS and toxicities were secondary endpoints. This trial was registered on the Clinical Trials website, reference number NCT 01752205.

### Patient population

Inclusion criteria consisted of histologically confirmed squamous cell carcinoma of the esophagus or esophagogastric junction; ineligibility for surgery; Eastern Cooperative Oncology Group (ECOG) performance status (PS) score 0–2; age ≥ 18 years old; life expectancy ≥ 12 weeks; no prior palliative therapy; at least one bidimensional measurable disease as defined by RECIST ver 1.1; adequate organ function for treatment; absolute neutrophil count (ANC) ≥ 1000 cells/mm^3^; platelets ≥ 100000 cells/mm^3^; estimated creatinine clearance ≥ 50 mL/min, or serum creatinine < 1.5× institutional upper limit of normal (ULN); bilirubin ≤ 1.5× ULN; AST (SGOT) ≤ 2.5× ULN (5.0× ULN if hepatic metastases); ALT (SGPT) ≤ 2.5× ULN (5.0× ULN if hepatic metastases); 12-lead electrocardiogram (ECG) with normal tracing or non-clinically significant changes that do not require medical intervention; QTc interval ≤ 470 ms and without history of Torsades de Pointes or other symptomatic QTc abnormality; LVEF (by MUGA or echocardiogram) of ≥ 50%. Exclusion criteria included prior thoracic radiotherapy or anti-EGFR treatment, uncontrolled brain metastasis, and other malignancies. All patients provided signed informed consent before participating in this study and were amenable to compliance with protocol schedules. The study was approved by the institutional ethics committee of the 307 Hospital of PLA.

Pre-enrollment examinations included physical examination, full blood counts, biochemistry tests, electrocardiograph, thoracic computed tomography (CT) and/or 18F-fluorodeoxy glucose positron emission tomography (18F-FDG PET), barium esophagram, cervical and abdominal ultrasound examination, endoscopic ultrasonography and bone scan. Disease was staged according to the 7th edition of the American Joint Committee on Cancer Staging System. During treatment, physical examination and full blood counts were repeated weekly, and biochemistry tests were repeated every two weeks.

### Treatment procedure

The treatment plan included administration of erlotinib (150 mg daily) for a period of 60 days beginning at the start of radiotherapy, and paclitaxel weekly along with intensity modulated conformal radiation therapy (IMRT). Gross tumor volume (GTV) was determined based on thoracic CT (or 18F-FDG PET if possible) and the endoscopy results. Clinical target volume (CTV) included the GTV and supraclavicular and mediastinal lymph node regions. The celiac lymph node region was also included in the CTV for tumors located in the lower third of the esophagus. Planning target volumes (PTV) were generated as the CTV plus 5 mm of margin. Planning objectives involved delivering 60 Gy in 30 fractions of 2 Gy per fraction over 6 weeks to obtain 95% PTV coverage. Dose modification for hematological toxicity was based on full blood counts taken within the 3 days before the start of weekly paclitaxel. Paclitaxel was omitted for grade 4 toxicity, and was reduced to 75% and 50% of the starting dose after the first and second occurrences of grade 3 toxicity, respectively. Sequential dose reduction of erlotinib to 100 mg/d was advised for second occurrences of grade 3 skin rash, and it was permanently discontinued after a third appearance.

### Immunohistochemical analysis

Levels of EGFR and VEGF expression were assessed immunohistochemically. All tissue samples were immersion fixed in 4% paraformaldehyde, embedded in paraffin, and sectioned at a thickness of 8 μm. Sections were probed first with anti-EGFR (1:100, rat polyclonal; Santa Cruz) or anti-VEGF (1:50, mouse monoclonal; Abcam) antibody overnight at 4°C, and then with secondary antibodies for 1 h at room temperature.

### EGFR mutation analysis

EGFR mutation was assessed using polymerase chain reaction (PCR)-direct sequencing. For gene mutation analysis, DNA was collected from primary esophageal tumor specimens using the phenol-chloroform extraction method after overnight digestion of the tissue using proteinase K. Mutation within the four tyrosine kinase domain exons (18–21) of EGFR that are frequently mutated in esophageal cancer was assessed using PCR-direct sequencing as previously reported [11]. All sequence variants were confirmed through independent PCR amplification and sequencing in both directions.

### Statistical analysis

In the only randomized trial designed to deliver adequate doses of PF with concurrent RT for ESCC (RTOG-8501), the two-year survival rate was 36%. We anticipated a survival rate of 46% in our study. The statistical design was intended to enable us to detect a response rate of at least 20%. It was calculated that a sample size of 20 patients was required.

Kaplan-Meier survival curves were estimated, and the log-rank test was applied to assess the differences in survival distributions related to EGFR expression or mutation and clinical variables. Statistical analysis was performed using SPSS software, version 20.0. All probability values were two-sided, and values of *p* < 0.05 were considered statistically significant.
